# Long-term air pollution exposure and cardio- respiratory mortality: a review

**DOI:** 10.1186/1476-069X-12-43

**Published:** 2013-05-28

**Authors:** Gerard Hoek, Ranjini M Krishnan, Rob Beelen, Annette Peters, Bart Ostro, Bert Brunekreef, Joel D Kaufman

**Affiliations:** 1 Institute of Risk Assessment Sciences, University of Utrecht, Utrecht, The Netherlands; 2 University of Washington, Seattle, WA, USA; 3 Institute of Epidemiology II, Helmholtz Zentrum München –German Research Center for Environmental Health, Neuherberg, Germany; 4 Air Pollution Epidemiology Section, Office of Environmental Health Hazard Assessment, State of California, Oakland, CA, USA; 5 Julius Center for Health Sciences and Primary Care, University Medical Center, Utrecht, The Netherlands

**Keywords:** Air pollution, Mortality, Motorized traffic, Cardiovascular, Respiratory, Particles

## Abstract

Current day concentrations of ambient air pollution have been associated with a range of adverse health effects, particularly mortality and morbidity due to cardiovascular and respiratory diseases. In this review, we summarize the evidence from epidemiological studies on long-term exposure to fine and coarse particles, nitrogen dioxide (NO_2_) and elemental carbon on mortality from all-causes, cardiovascular disease and respiratory disease. We also summarize the findings on potentially susceptible subgroups across studies. We identified studies through a search in the databases Medline and Scopus and previous reviews until January 2013 and performed a meta-analysis if more than five studies were available for the same exposure metric.

There is a significant number of new studies on long-term air pollution exposure, covering a wider geographic area, including Asia. These recent studies support associations found in previous cohort studies on PM_2.5_. The pooled effect estimate expressed as excess risk per 10 μg/m^3^ increase in PM_2.5_ exposure was 6% (95% CI 4, 8%) for all-cause and 11% (95% CI 5, 16%) for cardiovascular mortality. Long-term exposure to PM_2.5_ was more associated with mortality from cardiovascular disease (particularly ischemic heart disease) than from non-malignant respiratory diseases (pooled estimate 3% (95% CI −6, 13%)). Significant heterogeneity in PM_2.5_ effect estimates was found across studies, likely related to differences in particle composition, infiltration of particles indoors, population characteristics and methodological differences in exposure assessment and confounder control. All-cause mortality was significantly associated with elemental carbon (pooled estimate per 1 μg/m^3^ 6% (95% CI 5, 7%)) and NO_2_ (pooled estimate per 10 μg/m^3^ 5% (95% CI 3, 8%)), both markers of combustion sources. There was little evidence for an association between long term coarse particulate matter exposure and mortality, possibly due to the small number of studies and limitations in exposure assessment. Across studies, there was little evidence for a stronger association among women compared to men. In subjects with lower education and obese subjects a larger effect estimate for mortality related to fine PM was found, though the evidence for differences related to education has been weakened in more recent studies.

## Review

### Background

There is growing evidence of mortality effects related to long-term exposure (i.e., exposures of a year or more) to ambient air pollution [1-3]. Cardiovascular effects of short- and long-term exposure to particulate matter air pollution focusing on PM_2.5_ have recently been comprehensively reviewed [4,5]. Experimental and epidemiological studies in the recent decade have significantly increased our knowledge of mechanisms that could plausibly explain the associations observed in epidemiological studies between ambient air pollution and mortality [4].

Most studies have reported associations linked to particulate matter, often represented by the mass concentration of particles smaller than 10 μm (PM_10_) or 2.5 μm (PM_2.5_). In many urban areas, motorized traffic emissions are an important source of ambient particles and gaseous pollutants such as nitrogen oxides (NO_2_ and NO). Exposure contrasts related to traffic emissions are usually poorly represented by the concentration of PM_10_ or PM_2.5_, because of the high regional background concentration of these particle metrics from other sources [6,7]. However, there are more specific markers for traffic related air pollution, which include elemental carbon and ultrafine particles number [7-10]. Janssen and co-workers recently demonstrated that health impact assessments of traffic-related pollutants based upon PM_2.5_ seriously underestimated the health risks compared to an assessment based upon elemental carbon [7]. There is also growing evidence of health effects related to ultrafine particles [8,9]. Finally, the effects of coarse particles (the particle fraction between 2.5 and 10 μm) have attracted renewed attention [11]. Emission controls for road traffic have now substantially reduced tailpipe emissions, and therefore non-tailpipe emissions including engine crankcase emissions (combusted lubricating oil), road, tire and brake wear are becoming increasingly important. A recent study in the Netherlands found similar increases of concentrations in major roads compared to urban background for metals related to break and tire wear (Cu, Zn) as for soot and ultrafine particles which are due to tailpipe emissions [10] . In a review of the limited literature, coarse particles were associated with short-term effects on mortality and hospital admissions, but no evidence was found for long-term exposure effects [11]. The number of studies on long-term coarse particle exposure reviewed was small however at the time.

The aim of the current review is to evaluate the epidemiological evidence for cardiovascular and respiratory mortality effects of long-term exposure to fine particulate matter, including a meta-analysis. We focused on epidemiological studies of mortality, as experimental studies and mechanisms of effect have been discussed in detail previously [4]. The American Heart Association review [4] is updated with a significant number of new studies published in 2009 – 2012. We further include more pollutants in the review, specifically NO_2_, elemental carbon and coarse particles. We evaluated the findings on potentially susceptible subgroups across studies of PM_2.5_. In addition, we have included the studies on more specific cardiovascular causes of death, especially fatal myocardial infarction and stroke.

### Methods

We performed a search in the databases Medline and Scopus with the search terms air pollution, cohort, and mortality until January 2013. We supplemented the search with studies included in the review by Brook and co-worker [4] and by browsing the reference lists of identified papers. In case more than five studies were identified, we performed a meta-analysis. We tested for heterogeneity of cohort-specific effect estimates and obtained combined effects estimates, using random effects methods of DerSimonian and Laird [12]. The I^2^ statistic was calculated as a measure of the degree of heterogeneity across studies [13]. I^2^ ranges from 0 to 100% and can be interpreted as the variability of study-specific effect estimates attributable to true between study effects. From some studies multiple papers were available such as the Six Cities study [14-16]. In the meta-analysis we used only the most recent paper, which had longer follow-up. We only included studies in the quantitative meta-analysis that directly provided PM_2.5_ exposure estimates. For NO_2_ we only included studies which accounted for intra-urban spatial variation using e.g. dispersion models, land use regression models or spatial interpolation. We used STATA version 10 (Stata Corp, College Station, Texas) for meta-analysis. Effect estimates are presented as excess risks expressed per 10 μg/m^3^ contrast in exposure, except elemental carbon for which risks were expressed per 1 μg/m^3^.

#### PM_2.5_ and all-cause and cardiovascular mortality

**Figure 1:**
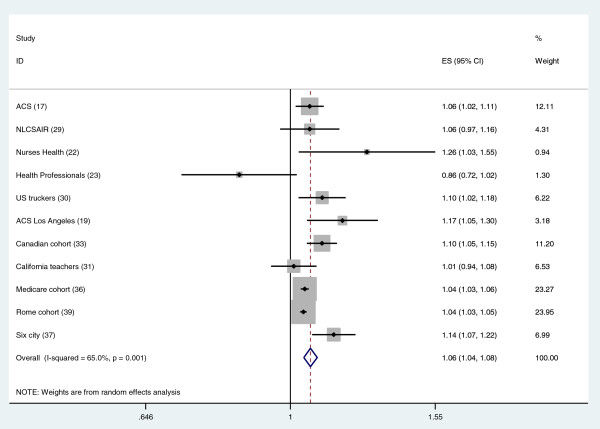
**Meta-analysis of the association between PM**_**2.5 **_**and all-cause mortality (Relative risk per 10 μg/m**^**3**^**).** Overall uses random effects.

**Figure 2:**
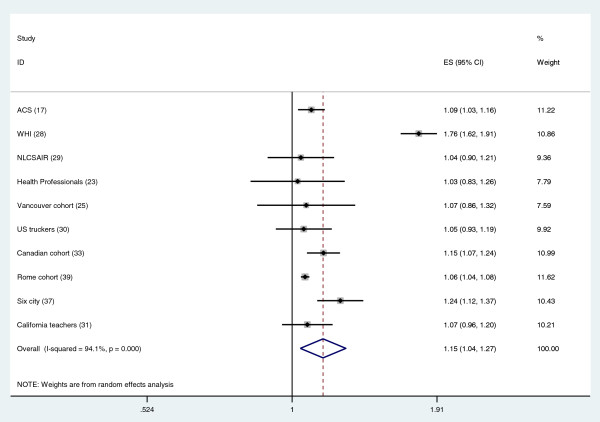
**Meta-analysis of the association between PM**_**2.5 **_**and cardiovascular mortality (Relative Risk per 10 μg/m**^**3**^**).** Overall uses random effects.

Table 1 and Figures 1 and 2 summarize the studies on long-term air pollution exposure and all-cause and cardiovascular mortality using PM_2.5_ or PM_10_ as exposure metric [14-39]. Most but not all studies report significant associations between PM_2.5_ and all-cause mortality. Since the publication of the authoritative American Heart Association Scientific Statement, sixteen new cohort studies were published between 2009 and January 2013. These studies were often performed in more selected groups e.g. female teachers [27,36] or male truck drivers [32]. The geographic range has also been expanded significantly with several new studies from Japan and China now published. Another tendency is the publication of large studies based upon large population samples (e.g. census), with often less information on confounding variables such as individual smoking habits. Large cohort studies have used neighborhood socio-economic status and co-morbidities strongly associated with smoking as proxies for actual smoking data [26,38]. Effect estimates differed substantially across studies, with most studies showing less than 10% increase in mortality for an increment of 10 μg/m^3^ PM_2.5_. The random effects summary estimate for the percent excess risk per 10 μg/m^3^ PM_2.5_ for all-cause mortality was 6.2% (95% CI: 4.1 – 8.4%). A formal test of heterogeneity was statistically significant, with an I^2^ value of 65% indicating moderate heterogeneity. I^2^ can be interpreted as the variability in effect estimates due to true between study variability and not chance [13]. The random effects summary effect estimate for cardiovascular mortality was 10.6% (95% CI 5.4, 16.0%) per 10 μg/m^3^. Thus, the overall effect estimates were larger for cardiovascular than for all-cause mortality. This pattern was found in most of the individual studies, with the exceptions being the Dutch cohort study [23], the US trucking industry cohort study [32] and a national cohort study from New Zealand [35]. Significant and large heterogeneity of effects was found across studies, with an I^2^ statistic of 61%. After excluding the Miller study [22], moderate heterogeneity remained (I^2^ = 40%). Overall, the new studies have supported an association between PM_2.5_ and mortality first identified in the US Six City and ACS studies. It is of interest to note that the weight of the ACS study in the combined effect estimate is 12% for all-cause mortality, documenting that the combined estimate does not rely on one or two studies. Furthermore, effect estimates from the three large population cohorts without individual smoking data [26,34,38] were not higher than those from the individual cohort studies. An important question is what the explanation is for the observed heterogeneity of effect estimates. Differences in study population, exposure assessment, pollution mixture, study period, outcome assessment, and confounder control could have contributed to these differences.

**Table 1:** **Summary of effect estimates (excess risk per 10 μg/m**^**3**^**) from cohort studies on particulate matter (PM**_**10 **_**or PM**_**2.5**_**) and mortality from all causes and cardiovascular diseases**

**Study**	**Study population**	**Follow-up period**	**Pollutant**	**Conc**^**a **^**(μg/m**^**3**^**)**	**Spatial scale**^**b**^	**% change in risk (95% CI) in mortality associated with a 10 μg/m**^**3 **^**increase PM**		**References**
						***All cause***	***Cardiovascular***^*c*^	
Harvard six cities	8111 adults in six US cities	1976 - 1989	PM_2.5_	18 (11–30)	City	13(4, 23)	18 (6, 32)	[15]
Harvard six cities	8096 adults in six US cities	1979 -1998	PM_2.5_	15 (10–22)	City	16 (7, 26)	28 (13,44)	[14]
Harvard six cities	8096 adults in six US cities	1974 - 2009	PM_2.5_	16 (11–24)	City	14 (7, 22)	26 (14, 40)	[16]
American Cancer Society (ACS) study	552, 800 adults from 51 US cities	1982 - 1989	PM_2.5_	18 (9–34)	City	26 (8, 47)	NA	[17]
ACS study	500,000 adults from 51 US cities	1982 -1998	PM_2.5_	18 (4)	City	6 (2, 11)	9 (3, 16)^c^	[18]
ACS sub-cohort study	22,905 subjects in Los Angeles area	1982 - 2000	PM_2.5_	(~9 – 27)	Zip code (Int)	17 (5, 30)	26 (1, 60)^c^	[19]
German cohort	4752 women in Ruhr area	1985 – 2003	PM_10_	44 (35–53)	Address (near)	12 (−9, 37)	52 (8, 114)	[20]
German cohort	4752 women in Ruhr and surrounding area	1985 - 2008	PM_10_	44 (35–53)	Address (near)	22 (6, 41)	61 (26, 104)	[21]
Women’s Health Initiative Observational Study	65,893 postmenopausal women from 36 US metropolitan areas	1994-1998	PM_2.5_	14 (3–28)	Zip code (near)	NA	76 (25,147)	[22]
Netherlands Cohort Study	120, 852 subjects from Netherlands	1987 -1996	PM_2.5_	28 (23–37)	Address (LUR)	6 (−3, 16)	4 (−10, 21)	[23]
Nurses’ Health Study	66,250 women from the US north eastern metropolitan areas	1992-2002	PM_10_	22 (4)	Address (LUR)	11 (1,23)	35 (3, 77)	[24]
Nurses’ Health Study	66,250 women from the US north eastern metropolitan areas	1992-2002	PM_2.5_	14 (6–28)	Address (LUR)	26 (2, 54)	NA	[25]
Medicare national cohort	13.2 million elderly Medicare recipients across the USA	2000 - 2005	PM_2.5_	13 (4)	Zip code (Mean)	4 (3, 6)^d^		[26]
California teachers study	45,000 female teachers	2002 -2007	PM_2.5_	18 (7–39)	Address (near)	6 (−4, 16)	19 (5, 36)^c^	[27]
Swiss national cohort	National census data linked with mortality	2000 - 2005	PM_10_	19 (>40)^e^	Address (Disp)	NA	−1 (−3, 0)	[28]
Health professionals follow-up study	17,545 highly educated men in the midwestern and northeastern US	1989 – 2003	PM_2.5_	18 (3)	Address (LUR)	−14 (−28,2)	3 (−17, 26)	[29]
Vancouver cohort	452,735 Vancouver residents 45–85 yr	1999 – 2002	PM_2.5_	4 (0 – 10)	Address (LUR)	NA	7 (-14, 32)	[30]
China nat. hypertension survey	70,497 men and women	1991 - 2000	TSP	289 (113–499)	City	0.3 (0, 1)	1 (0, 2)	[31]
US trucking industry cohort	53,814 men in the US trucking industry	1985 -2000	PM_2.5_	14 (4)	Address (near)	10 (3, 18)	5 (−7, 19)	[32]
Chinese retrospective cohort study	9,941 adults from five districts of Shenyang city	1998 -2009	PM_10_	154 (78–274)^f^	District (mean)	53 (50, 56)	55 (51, 60)	[33]
Canadian national cohort	2.1 million nonimmigrant Canadians . > 25 yr	1991 - 2001	PM_2.5_	9 (2 – 19)	Enumeration area, N = 45710 (satellite)	10 (5, 15)	15 (7, 24)	[34]
New Zealand Census mortality study	1.06 million adults in urban areas from 1996 census	1996 -1999	PM_10_	8 (0 – 19)	Census tract (Disp)	7 (3, 10)	6 (1, 11)	[35]
California teachers study	101,784 female teachers	1997- 2005	PM_2.5_	16 (3–28)	Address (Inter)	1 (−5, 9)	7 (−5, 19)	[36]
Nippon data cohort	7,250 adults > 30 yr throughout Japan	1980 - 2004	PM_10_	<27 - > 43	District (near)	−2 (−8, 4)	−10 (−19, 0)	[37]
Rome longitudinal study	1,265,058 adults from Rome	2001 - 2010	PM_2.5_	23 (7 – 32)	Address (DISP, 1 km grid)	4 (3, 5)	6 (4, 8)	[38]

^a^ Mean with minimum – maximum in parentheses (μg/m^3^). One number in parentheses is standard deviation.

^b^ Spatial scale of exposure assignment, in parentheses exposure assignment method. City = average of monitors within the city; Near = nearest monitor concentration; LUR = land use regression; Disp = dispersion modeling; Inter = interpolation.

^c^ Cardio-pulmonary mortality reported if cardiovascular mortality not available.

^d^ Combining the estimates from the three regions of the USA.

^e^ Median and 90th percentile reported.

^f^ Very high pollution levels that changed significantly during follow-up changing the ranking of the five districts.

Studies adjusted for individual smoking except references [26,28,30,34,38,56].

#### Effect modification

Differences in the fraction of *susceptible subjects* may have contributed to the observed differences. Brook [4] suggested that women might be more susceptible to ambient air pollution. The studies with higher PM effect estimates, particularly the WHI-study have indeed been performed in women only. However, it is problematic to draw conclusions about susceptible subgroups based upon *between-study* comparisons as multiple factors differ between studies. A comparison of PM effect estimates between men and women *within* studies does not provide clear evidence that women have a stronger response (Table 2). The findings from the AHSMOG are difficult to interpret, with higher effects in men in the larger earlier study [40] and larger effects in women in the smaller cohort with longer follow-up [41]. The larger effect estimate for BC for men in a Canadian study [30] has to be interpreted with care, because of the lack of data on a variety of important covariates, including individual smoking data, though the authors argue that smoking likely has not confounded the associations with mortality. In the French PAARC study, effect estimates for the evaluated pollutants (TSP, BS and NO_2_) were similar among men and women [42]. There is also only weak evidence that effect estimates are larger among never-smokers, though in all evaluated studies a (borderline) significant association was found in never-smokers (Table 2). Associations in current smokers were more variable across the studies, consistent with the larger ‘noise’ generated by smoking. In all four studies, PM_2.5_ effect estimates were higher for those with the lowest education and there was little indication of an association in those with higher education. The absence of an association in the (highly educated) Health professionals study [29] is consistent with this observation. In contrast, in the French PAARC study, effect estimates for Black Smoke were very similar across educational strata, with significant effect also found in those with a university degree [42]. Furthermore the PM_2.5_ effect estimates (excess risks) in an extended analysis of the ACS differed less than originally reported: 8.2%, 7.2 and 5.5% per 10 μg/m^3^ for subjects with low, medium and high education respectively [43]. If confirmed in further studies, it is likely that multiple life style related factors may play a role in the stronger effects observed in less-educated subjects. These may include dietary factors such as lower fruit and anti-oxidant intake [23], higher risk of obesity or other pre-existing diseases, higher actual exposures than assumed in the studies, lack of air conditioning and possibly interaction with other risk factors such as poorer housing conditions e.g. moisture.

**Table 2:** **Effect modification of the effect (excess risk per 10 μg/m**^**3**^**) of PM**_**2.5 **_**on cardiovascular mortality**

**Subgroup**	**ACS [**[18]**]**^**a**^	**NLCS [**[23]**]**	**Harvard six city [**[43]**]**	**Nurses health [**[24]**]**	**WHI [**[22]**]**	**AHSMOG [**[40]**]**	**AHSMOG [**[41]**]**
***Sex***							
Men	5 (0, 11)	3 (−5, 12)^b^	33 (8, 63)^a^	NA	NA	4 (−3,11)	−10 (−−24, 5)
Women	6 (0, 12)	7 (0, 14)	20 (−6, 53)			−3 (−9, 2)	42 (6, 90)
*Smoking status*							
Never	6 (1, 12)	13 (−4, 32)	36 (2, 82)	83 (20, 179)	18 (−1, 40)	NA	NA
Former	5 (0, 11)	−4 (−17, 13)	29 (−3, 72)	22 (−18, 83)	21 (1, 52)		
Current	4 (−2, 11)	3 (−10, 19)	35 (94, 74)	−12 (−48, 48)	68 (6, 166)		
*Education*							
Low	11 (6, 18)	20 (−10, 70)^a^	45 (13, 85)		40 (11, 75)	NA	NA
Medium	6 (1, 13)	2 (−16, 24)	30 (−2,73)		33 (14, 55)		
High	1 (−3, 6)	−10 (−35, 20)	−3 (−29, 34)		11 (−6, 31)		
*Body mass index*							
Non-Obese	NA	NA	NA	8 (−24, 52)	−1 (−10, 29)^c^	NA	NA
Obese				99 (23, 222)	35 (12, 64)^c^		

^a^ Read from graph.

^b^ natural-cause mortality.

^**c**^ for BMI < 22.5, continuous trend observed NA = not available.

In two studies, PM_2.5_ effect estimates were substantially higher among subjects with high body mass index [22,24].

It is likely that subject characteristics might explain part of the variability of air pollution effect estimates across studies where subgroup analyses are limited by power to detect differences. Hence, further research is required to study the effects of air pollution on women, smokers, obese participants, and diabetes mellitus with better measurement of the exposures. Gene-environment interactions have been shown for the (short-term) air pollution effects on inflammation markers [44,45] Inflammation likely plays an important role in the mechanism of cardiovascular events [3,4]. Gene-environment interactions have not yet been studied in the framework of mortality cohort studies.

#### Exposure issues

One of the important sources of variability of effect estimates between studies is likely related to exposure definition and misclassification. While the most important environmental predictor to consider is actual individual-level *exposure* to ambient particles, which presumably drives the health effects, most studies have used outdoor concentrations at sites distant to the participant’s precise location. The use of outdoor exposures leads to exposure misclassification. In the cohort studies, exposure has been characterized by the outdoor concentration at the city level based upon central site monitoring or the nearest monitor, or modeling at the individual address. Table 1 shows that the spatial scale of assessment and exposure assessment method varied significantly across studies, probably contributing to differences in effect estimates. Differences in pollution range across studies (Table 1) may have contributed as well. These exposure estimates do not take into account time activity patterns such as time spent in the home or in traffic and factors affecting infiltration of particles indoors. There is a large literature documenting the importance of air exchange rate on infiltration of particles indoors. Importantly, these factors may differ between homes within a study area and between study areas in different climates. In a study of short-term effects, PM10 effects on hospital admissions were larger in US cities with lower% of air conditioning, related to higher particle infiltration rates [46]. The impact of air conditioning use has not been investigated yet in the framework of cohort studies. In the Multiethnic study of Atherosclerosis Air study, indoor-outdoor measurements have been performed to adjust the exposure estimates [47,48] and each participant provides time-activity information to weight exposures between time spent indoors and outdoors. Evidence for the importance of time activity patterns was obtained in the US truckers study, showing higher ambient PM_2.5_ effect estimates in the population excluding long-haul drivers who spend more time away from home [32]. Other factors could however also explain the higher effect estimated after excluding long-haul drivers. In the WHI study, effect estimates tended to be higher for subjects spending more than 30 minutes outdoors [22]. In a validation study in the Netherlands, the contrast of personal soot exposure for adults living on a major road compared to those living at a background location, was larger for those spending more time at home [49]. Because of the reliance on ambient exposure estimates, it is not surprising that some heterogeneity in effect estimates across studies is found.

Differences in *particle composition* or contributing sources very likely explain some of the heterogeneity in effect estimates, as was observed for short-term mortality and hospital admission studies of PM_2.5_ and PM_10_[50-53]. For a comprehensive review we refer to the recent evaluation made by the World Health Organization (http://www.euro.who.int/en/what-we-do/health-topics/environment-and-health/air-quality/publications/2013/review-of-evidence-on-health-aspects-of-air-pollution-revihaap). Particle composition effects have not been systematically investigated in cohort studies with the exception of the California teacher’s study [27]. In a recent review it was shown that on a per microgram per m^3^ basis, mortality effect estimates were about 10 times larger for EC than for PM_2.5_[7]. Hence, in locations with higher levels of primary combustion particles we could expect higher PM_2.5_ effects. In the next section, evidence on EC is further discussed.

A further important issue is for which *period exposure* is characterized. Air pollution data may not be available for the entire follow-up period. As an example in the ACS study, PM_2.5_ data were available at the start and end of follow-up [18]. When significant (often downward) trends in pollution occur with changing (often decreasing) spatial contrasts in the study, bias may occur in the estimated association between pollution and mortality. The follow-up study from the Harvard Six City study [14] and two studies in potentially at-risk populations [54,55] suggested that the relevant exposure for cardiovascular effects may be the exposure in the past few years. These authors conclude that it does not take decades to bridge the gap between the short- and long-term exposure effect estimates, consistent with the effect of intervention studies showing reductions in mortality in the year after the intervention [54,55]. These studies [54,55] have made use of long-term temporal contrast within cities adjusting for secular trends. PM effect estimates were similar to the previously discussed studies exploiting spatial contrasts.

A further *temporal* issue in studies that use land use regression models for exposure assessment is that these models often are based upon current measurement campaigns and linked to health outcomes that occurred in the past. Three studies in the Netherlands, Rome (Italy) and Vancouver (Canada) have shown that for periods of about 10 years current LUR models predicted historic spatial contrasts well [56-58]. Even when concentrations have decreased over time, spatial contrasts often remain stable. Spatial contrasts may not be stable in areas with rapid economic development as indicated in one of the Chinese cohort studies in which the ranking of study areas changed during follow-up [33,59]. Even when the ranking of subjects is not changed, the quantitative spatial contrast in a study area may have changed, e.g. because the difference between major roads and background locations has decreased in time. Changed spatial contrasts will affect the estimated slope of the mortality pollution association [18,56]. Moving of subjects may further complicate the assessment.

An important question to address for the traffic pollution studies is potential confounding by *road traffic noise*, which has been shown to be related to cardiovascular disease including MI as well. A few studies have attempted to disentangle traffic-related air pollution and noise [60-62]. These studies found moderate correlations between air pollution and noise. The three studies differed somewhat in their findings of independent air pollution and noise effects. More work is needed in this area.

#### Coarse particles and elemental carbon

Table 3 presents studies that have used elemental carbon or coarse PM as the exposure metric. Table 3 illustrates that there is no evidence that long-term exposure to coarse PM is related to mortality. In three of the four cohort studies that reported no significant association with coarse PM, significant associations with PM_2.5_ were found [18,25,63]. However, exposure assessment for coarse particles is more challenging than for PM_2.5_ because of the influence of local sources, hence central site monitors are likely to have greater errors in representing residential concentrations. It is therefore possible that with more spatially resolved exposure assessment methods such as land use regression models or dispersion models, potential long-term exposure effects will be detected. The California Teacher’s study did not evaluate coarse PM and did not find significant associations between all-cause mortality and elemental concentrations of Si, Fe and Zn, elements abundant in coarse particles, but did report an association between Si and ischemic heart disease [27].

**Table 3:** **Summary of effect estimates (excess risk per 10 μg/m**^**3**^**) from cohort studies on coarse particulate matter and elemental carbon (per 1 μg/m**^**3**^**) and mortality from all causes and cardiovascular diseases**

**Study name**	**Study design**	**Follow-up period**	**Pollutant**	**Conc**^**a**^** (μg/m**^**3**^**)**	**Spatial scale**^**b**^	**% change in risk (95% CI) in mortality**		**References**
						***All causes***	***Cardiovascular***^*c*^	
*Coarse PM*								
ACS study	500,000 adults 51 US cities	1982 - 1998	PM_2.5–15_	19 (6)	City	1 (−2 3)	2 (−2, 5)*	[18]
AHSMOG study	3769 California seventh-day Adventists	1977 – 1992	PM_2.5–15_	27 (4 – 44)	Address (Inter)	5 (−8, 20)	NA	[63]
Nurses’ Health Study	66,250 women from US north eastern metropolitan areas	1992- 2002	PM_2.5–10_	8 (0 – 27)	Address (LUR)	3 (−11, 18)	NA	[25]
Health professionals follow-up study	17,545 highly educated men in the midwestern and northeastern US	1989 – 2003	PM_2.5–10_	10 (3)	Address (LUR)	−10 (−22, 4)	8 (−10, 29)	[29]
*EC*								
Netherlands Cohort Study	120, 852 subjects from Netherlands	1987 - 1996	BS^e^	17 (9–36)	Address (LUR)	5 (0, 11)	4 (−5, 13)	[23]
ACS study (extended)	500,000 adults 51 US cities	1982 – 1998	EC	IQR = 0.31	City	6 (1, 11)	11 (3, 19)	[64]
Worcester MI survivors	3,895 MI patients	1995 - 2005	EC	0.4 (0.1 – 0.9)	Address (LUR)	2 (−7, 11)^d^	NA	[65]
						15 (3, 29)		
Vancouver cohort	452,735 Vancouver residents 45–85 yr	1999 – 2002	BC	1.5 (0–5)	Address (LUR)	NA	6 (3, 9)	[30]
PAARC	14,284 adults in 24 French areas	1974 – 1998	BS	44 (18–77)	Address (near)	7 (3, 10)	5 (−2, 12)	[42]
Veteran’s study	70,000 male US veterans	1997 – 2001	EC	0.6 (0.1 – 2.0)	County (mean)	18 (5, 33)	NA	[66]
California teachers study	45,000 female teachers	2002 -2007	EC	1.1 (0.2 – 2.4)	Address (near)	3 (−11,19)	11 (−9, 36)	[27]
Two Scotch cohorts	15, 402 and 7,028 adults from West-central and central Scotland	1972 - 1998 1970 - 1998	BS	19	LUR + temporal	5 (1,9)	7 (0, 13)	[67]

^a^ Mean with minimum – maximum in parentheses (μg/m^3^). One number in parentheses is standard deviation.

^b^ Spatial scale of exposure assignment, in parentheses exposure assignment method. City = average of monitors within the city; Near = nearest monitor concentration; LUR = land use regression; Disp = dispersion modeling; Inter = interpolation.

^c^ Cardio-pulmonary mortality reported if cardiovascular mortality not available.

^d^ HRs for first two years after MI and after the first two years of survival.

^e^ BC (Black Carbon), BS (Black Smoke) and EC (Elemental carbon) are different markers used to assess soot. Increases consistent with a 1 μg/m^3^ increase in EC were used [7].

Studies adjusted for individual smoking except references [26,28,30,34,38,56].

Consistently, the summary estimate for PM_10_ was smaller than for PM_2.5_ with a summary effect estimate per 10 μg/m^3^ of 3.5% (95% CI 0.4%, 6.6%) with significant heterogeneity (I^2^ = 69%) of the studies included in Table 1, excluding the because of changing spatial patterns difficult to interpret Chinese retrospective study [33]. The PM_10_ analysis was added as several studies only report PM_10_.

Effect estimates for EC were very consistent across studies [23,27,30,42,64-67]. The random effects summary estimate for all-cause mortality per 1 μg/m^3^ EC was 6.1% (95% CI 4.9%, 7.3%), with highly non-significant heterogeneity of effect estimates (I^2^ = 0%). Most of the included studies assessed EC exposure at the city-scale [27,64] which represents variation in city background but does not account for small-scale variation related to proximity to major roads. Many studies have documented significant intra-urban contrasts for EC, related to especially major roads [7]. Most likely EC and NO_2_ should be considered representatives of the complex mixture of traffic-related air pollution, rather than the only components causally associated with mortality.

There is fairly consistent evidence of associations of mortality with nitrogen dioxide (Table 4). The random effects summary estimate for all-cause mortality per 10 μg/m^3^ for NO_2_ was 5.5% (95% CI 3.1%, 8.0%), with significant and large heterogeneity of effect estimates (I^2^ = 73%). In this analysis, the Chinese study [33] was not included as exposure was assessed at the district level. Inclusion of the essentially null findings of the ACS study-excess risk of 0.3% (95% CI −0.8, 1.3%)- resulted in an only slightly smaller combined estimate of 4.7% (95% CI 2.4, 7.1%). In the ACS study, intra-urban variation was also not accounted for. As traffic-related air pollution varies on a small spatial scale, it is even more critical to assess exposure on a fine spatial scale such as the residential address than for PM_2.5_.

**Table 4:** **Summary of cohort studies on NO**_**2 **_**and mortality from all causes and cardiovascular diseases (excess risk per 10 μg/m**^**3**^**)**

**Study name**	**Study population**	**Follow-up period**	**Pollutant**	**Conc**^**a **^**(μg/m**^**3**^**)**	**Spatial scale**^**b**^	**% change in risk (95% CI) in mortality per 10 μg/m**^**3**^		**References**
						***All causes***	***Cardiovascular***	
Oslo cohort	16,209 men in Oslo, Norway	1972 – 1998	NO_x_	11 (1 – 168)	Address (DISP)	8 (6,11)	NA	[68]
Netherlands Cohort Study	120, 852 subjects from Netherlands	1987 -1996	NO_2_	37 (15–67)	Address (LUR)	8 (0, 16)	7 (−6, 21)	[23]
German cohort	4752 women in Ruhr and surrounding area	1985 – 2003	NO_2_	39 (20 – 60)	Address (near)	11 (1,21)	36 (14, 63)	[20]
German cohort	4752 women in Ruhr and surrounding area	1985 – 2008	NO_2_	39 (20 – 60)	Address (near)	11 (4,18)	32 (18, 47)	[21]
PAARC	14,284 adults in 24 French areas	1974 – 1998	NO_2_	20 (12 – 32)	Address (near)	14 (3, 25)	27 (4, 56)	[42]
China nat. hypertension survey	70,497 men and women	1991 - 2000	NOx	50 (20 – 122)	City	2 (0, 3)	2 (1, 4)	[31]
Vancouver cohort	452,735 Vancouver residents aged 45–85 yr	1999 – 2002	NO_2_	32 (15 – 58)	Address (LUR)	NA	5 (1, 9)	[30]
DCH	52,061 adults in Copenhagen and Arhus	1993 - 2009	NO_2_	17 (11 – 60)	Address (DISP)	8 (2, 13)	15 (3,27)	[69]
US trucking industry cohort	53,814 men in the US trucking industry	1985 -2000	NO_2_	28 (14)	Address (LUR)	5 (3, 7)	4 (0, 8)	[32]
Chinese retrospective cohort study	9,941 adults from five districts of Shenyang city	1998 -2009	NO_2_	46 (18–78)	District (mean)	145 (134, 158)	146 (131, 163)	[33]
Rome longitudinal study	684,000 adults from Rome	2001 - 2006	NO_2_	45 (11)	Address (LUR)	4 (3, 5)	NA	[56]
California Teachers study	101,784 female teachers	1997 -2005	NO_2_	67 (10 – 134)	Address (Inter)	−3 (−9, 4)	−2 (−12, 9)	[36]
Shizuoka elderly cohort	13,444 adults > 65 yr	1999 - 2006	NO_2_	25 (−19, 75)	Address (LUR)	2 (−4, 8)	15 (3, 28)	[70]
Ontario tax cohort	205, 440 adults in Toronto, Hamilton,Windsor	1982 – 2004	NO_2_	43 (8), 31 (6), 24 (5)^c^	Address (LUR)	NA	8 (5, 11)	[71]
Rome longitudinal study	1,265,058 adults from Rome	2001 - 2010	NO_2_	44 (13–75)	Address (LUR)	3 (2, 3)	3 (2, 4)	[38]

^a^ Mean with minimum – maximum in parentheses (μg/m^3^). One number in parentheses is standard deviation.

^b^ Spatial scale of exposure assignment, in parentheses exposure assignment method. City = average of monitors within the city; Near = nearest monitor concentration; LUR = land use regression; Disp = dispersion modeling; Inter = interpolation.

^c^ Mean (IQR) per city.

Studies adjusted for individual smoking except references [26,28,30,34,38,56].

#### Specific cardiovascular causes of death

Table 5 shows associations between ambient air pollution and mortality from ischemic heart disease or myocardial infarction (MI), including studies based upon death certificates, more detailed studies using registry data, or ideally cohort studies with epidemiological review of medical records, allowing more precise identification of disease incidence. Several case–control studies based upon M.I. registries or epidemiological studies with clinical review have found associations between NO_2_ and fatal M.I. but not non-fatal M.I. [72-74]. Thus far, the finding of associations for fatal MI only was interpreted as an evidence that air pollution particularly affects the frail, or acts to aggravate a disease progression caused by other factors. On the other hand, it is also possible that the outcomes of ischemic heart diseases are misclassified and combined as composite outcomes, where fatal outcomes are captured more precisely [75]. Although there is increasing evidence that air pollution is associated with markers of early atherosclerosis, it is possible that air pollution will affect the underlying biological processes that predispose to atherothrombosis (which leads to MI and stroke) compared to atherosclerosis [76,77]. Another explanation is that the type of outcomes affected by pollution are those that have higher case-fatality rates (e.g., arrhythmic sudden death has higher case-fatality rate than overall MI).

**Table 5:** Summary of the studies on particulate matter and NO_2_ and mortality from specific cardiovascular diseases (excess risk per 10 μg/m^3^)

**Study name**	**Pollutant**	**Conc**^**a **^**(μg/m**^**3**^**)**	**Spatial scale**^**b**^	**% change in risk (95% CI) in mortality associated with a 10 μg/m**^**3 **^**increase**			**References**
				***IHD mortality***	***M.I mortality***	***Cerebrovascular mortality***	
ACS study	PM_2.5_	17 (5)	City	18 (14, 23)	NA	2 (−5, 10)	[39]
Oslo cohort	NO_x_	11 (1 – 168)	Address (DISP)	8 (3, 12)	NA	4 (−6, 15)	[68]
Women’s Health Initiative Study	PM_2.5_	14 (3–28)	Zip code 5 (near)	76 (25,147)	NA	NA	[22]
Netherlands Cohort Study	BS	17 (9–36)	Address (LUR)	1 (−17, 22)	NA	39 (−1, 94)	[23]
Nurses’ Health Study	PM_10_	22 (4)	Address (LUR)	35 (3, 77)	NA	NA	[24]
Nurses’ Health Study	PM_2.5_	14 (6–28)	Address (LUR)	NA	102 (7, 278)	NA	[25]
California teachers study	PM_2.5_	18 (7–39)	Address (near)	55 (24, 93)	NA	NA	[27]
Swiss national cohort	PM_10_	19 (>40)^c^	Address (Disp)	−1 (−3, 0)	NA	−1 (−2, 0)	[28]
Health professionals follow-up study	PM_2.5_	18 (3)	Address (LUR)	−2 (−30, 35)	NA	NA	[29]
Canadian national cohort	PM_2.5_	9 (2 – 19)	Enumeration area, N = 45710 (satellite)	30 (18,43)	NA	4 (−7, 16)	[34]
Californian Teachers study	PM_2.5_	16 (3–28)	Address (Inter)	20 (2, 41)	NA	16 (−8, 46)	[36]
Shizuoka elderly cohort	NO_2_	25 (−19, 75)	Address (LUR)	27 (2, 58)	NA	9 (−6, 27)	[70]
Nippon data cohort	PM_10_	<27 - > 43	District (near)	−8 (−27, 17)	NA	−14 (−26,1)	[37]
DCH	NO_2_	17 (11 – 60)	Address (Disp)	7 (−9, 26)	NA	6 (−14, 32)	[69]
Ontario Tax cohort	NO_2_	43 (8), 31 (6), 24 (5)^c^	Address (LUR)	9 (4, 14)	NA	−4 (−10, 5)	[71]
Rome longitudinal study	PM_2.5_	23 (7 – 32)	Address (DISP, 1 km grid)	10 (6, 13)	NA	8 (4, 13)	[38]
*M.I. registry studies*							
Stockholm	NO_2_	14 (3 – 32)	Address (DISP)	NA	15 (−1, 33)	NA	[72]
Rome residents	NO_2_	(<30 - > 60)	Census block (LUR)	NA	7 (2, 12)	NA	[73]
Stockholm residents	NO_2_	12 (2 – 33)	Address (DISP)	NA	8 (5, 11)	NA	[74]

IHD = ischemic heart disease; MI = myocardial infarction. Fatal MI reported for registry studies. NA = not available.

^a^ Mean with minimum – maximum in parentheses (μg/m^3^). One number in parentheses is standard deviation.

^b^ Spatial scale of exposure assignment, in parentheses exposure assignment method. City = average of monitors within the city; Near = nearest monitor concentration; LUR = land use regression; Disp = dispersion modeling; Inter = interpolation.

^c^ Median and 90th percentile reported.

Studies adjusted for individual smoking except references [26,28,30,34,38,56].

Fewer studies have evaluated cerebrovascular mortality. In the Dutch cohort study and in the Women’s Health Initiative Study, a strong association was found [22,23]. In contrast, in the ACS study, the Norwegian cohort, and the Swiss national cohort study no association was found [28,39,68]. It is possible that poorer recording of cerebrovascular mortality on death certificates has contributed to these inconsistencies. There is also some evidence from ecological studies that air pollution may contribute to stroke mortality [78,79].

Two studies have reported significant associations between particulate matter air pollution and dysrhythmia, heart failure and cardiac arrest combined [39,60]. These results are based upon smaller numbers of events, and require large cohort studies for further verification. The results are consistent with several studies documenting significant associations between short-term PM or NO_2_ exposure and mortality due to heart failure and dysrhythmia and defibrillator discharges [4,80].

#### Air pollution and respiratory mortality

Table 6 shows the effect estimates for respiratory mortality. In the two first US cohort studies, no association between PM_2.5_ and respiratory mortality was found [15,17]. In contrast to the findings of these US studies, strong associations were found in the Dutch cohort study [23], a Norwegian study [68] and a Chinese study [59]. The random effect pooled estimate per 10 μg/m^3^ for PM_2.5_ was 2.9% (95%CI −5.9, 12.6%), highly non-significant. The heterogeneity across studies was statistically significant with an I^2^ statistic of 59%, indicating moderate heterogeneity. Associations for PM were weaker in the Dutch and Chinese cohort study than with NO_2_ or NO_x_. Respiratory mortality may be more related to primary traffic-related pollutants than with long-range transported particles, though further work is needed to test this hypothesis. The smaller number of deaths due to respiratory disease compared to cardiovascular diseases, contributed to larger confidence intervals within individual studies and larger variability of the main effect estimates across studies. In time series studies including several large multi-city studies in the USA and Europe, significant associations between daily variations in PM and respiratory mortality were found [1-4]. Expressed per 10 μg/m^3^ PM excess risks of about 1% are typically reported for short-term exposures, larger than for all-cause mortality [1-4]. In contrast to cardiovascular disease, current evidence therefore does not suggest an additional risk from long-term exposure, possibly related to mortality displacement [2,3]. More studies are needed to evaluate long-term exposures on respiratory mortality more thoroughly.

**Table 6:** **Summary of the studies on air pollution and mortality from all respiratory disease (excess risk per 10 μg/m**^**3**^**)**

**Study Name**	**Pollutant**	**Conc**^**a **^**(μg/m**^**3**^**)**	**Spatial scale**^**b**^	**% change in risk (95% CI) in mortality per 10 μg/m**^**3**^	**References**
AHSMOG	PM_10_	51 (17)	Address (Inter)	6 (−1, 15)	[40]
ACS study	PM_2.5_	17 (5)	City	−8 (−14, -2)	[39]
Oslo cohort	NO_x_	11 (1 – 168)	Address (DISP)	16 (6, 26)	[68]
Harvard six cities	PM_2.5_	15 (10–22)	City	8 (−21, 49)	[14]
Netherlands Cohort Study	PM_2.5_	28 (23–37)	Address (LUR)	7 (−25, 52)	[23]
Netherlands Cohort Study	NO_2_	37 (15–67)	Address (LUR)	12 (0, 26)	[23]
California Teachers study	PM_2.5_	18 (7–39)	Address (near)	3 (−20, 34)	[27]
China national. hypertension survey	NO_x_	50 (20 – 122)	City	3 (0, 6)	[31]
China national. hypertension survey	TSP	289 (113 – 499)	City	0.3 (−1,1)	[31]
US truckers study	PM_2.5_	14 (4)	Address (near)	20 (−9, 60)	[32]
US truckers study	NO_2_	28 (14)	Address (LUR)	15 (1,31)	[32]
California Teachers study	PM_2.5_	16 (3–28)	Address (Inter)	21 (−3, 52)	[36]
New Zealand Census study	PM_10_	8 (0 – 19)	Census tract (Disp)	14 (5, 23)	[35]
Shenyang cohort study	PM_10_	154 (78 – 274)	District (mean)	67 (60, 74)	[59]
Shenyang cohort study	NO_2_	46 (18–78)	District (mean)	197 (169, 227)	[59]
Shizuoka elderly cohort	NO_2_	25 (−19, 75)	Address (LUR)	19 (2, 38)	[70]
Two Scotch cohorts	BS	19	LUR + temporal	11 (−3, 28)	[67]
Rome longitudinal study	PM_2.5_	23 (7 – 32)	Address (DISP, 1 km grid)	3 (−3, 8)	[38]

^a^ Mean with minimum – maximum in parentheses (μg/m^3^). One number in parentheses is standard deviation.

^b^ Spatial scale of exposure assignment, in parentheses exposure assignment method. City = average of monitors within the city; Near = nearest monitor concentration; LUR = land use regression; Disp = dispersion modeling; Inter = interpolation.

Studies adjusted for individual smoking except references [26,28,30,34,38,56].

## Conclusions

There is a significant number of new studies on long-term air pollution exposure, covering a wider geographic area, including Asia. These recent studies support associations found in previous cohort studies on PM_2.5_. The pooled effect estimate expressed as excess risk per 10 μg/m^3^ increase in PM_2.5_ exposure was 6% (95% CI 4, 8%) for all-cause and 11% (95% 5, 16%) for cardiovascular mortality. Long-term exposure to PM_2.5_ was more associated with mortality from cardiovascular disease (particularly ischemic heart disease) than from non-malignant respiratory diseases (pooled estimate 3% (95% CI −6, 13%)). Significant heterogeneity in PM_2.5_ effect estimates was found across studies, likely related to differences in particle composition, infiltration of particles indoors, population characteristics and methodological differences in exposure assessment and confounder control. All-cause mortality was significantly associated with elemental carbon (pooled estimate per 1 μg/m^3^ 6% (95% CI 5, 7%)) and NO_2_ (pooled estimate per 10 μg/m^3^ 5% (95% CI 3, 8%)), both markers of combustion sources. There was little evidence for an association between long term coarse particulate matter exposure and mortality, possibly due to the small number of studies and limitations in exposure assessment. Across studies, there was little evidence for stronger association among women compared to men. Subjects with lower education and obese subjects experienced larger mortality effect related to fine PM, though the evidence for differences related to education has been weakened in more recent studies.

Our review suggests several specific research questions. Research into the reasons for the heterogeneity of effect estimates would be extremely useful for health impact assessment. Better exposure assessment including spatially resolved outdoor exposures and more chemically speciated PM might in part be able to resolve the observed heterogeneity. Chemical speciation would allow assessing particles from different sources e.g. particles from combustion sources and non-tailpipe emissions separately, a question clearly relevant for air pollution control policy. Specific attention to motorized traffic emissions is important because (road) traffic is an important source of ambient air pollution. More work on coarse particles and at the other side of the particle size spectrum, ultrafine particles is needed. Ongoing new research in the USA in the Multi-Ethnic study of Atherosclerosis and Air pollution (MESA-AIR) and the European Study of Cohorts for Air Pollution Effects (ESCAPE) that use large cohorts and state-of the art spatially-resolved exposure methods will likely contribute significant new answers in the near future to these questions.

## Abbreviations

ACS: American Cancer Society study; BS: Black Smoke; BC: Black Carbon; CI: Confidence interval; EC: Elemental Carbon; NO2: Nitrogen dioxide; NOx: Nitrogen oxides; PM: Particulate matter; PM2.5: Particles smaller than 2.5 μm; PM10: Particles smaller than 10 μm; TSP: Total suspended particles.

## Competing interests

None of the authors has a competing interest.

## Authors’ contributions

GH, RMK, RB, AP, BO, BB and JK have contributed to the definition of the scope of the review, identification of studies and interpretation of results. GH drafted the text. GH, RMK, RB, AP, BO, BB and JK provided critical comments and approved the final manuscript.
